# Burnout Among Hospitalists During the Early COVID-19 Pandemic: a National Mixed Methods Survey Study

**DOI:** 10.1007/s11606-023-08309-x

**Published:** 2023-07-28

**Authors:** Anne Becker, Erin E. Sullivan, Luci K. Leykum, Roger Brown, Mark Linzer, Sara Poplau, Christine Sinsky

**Affiliations:** 1 Hennepin Healthcare, Minneapolis, MN USA; 2 Sawyer Business School, Suffolk University, Boston, MA USA; 3 Harvard Medical School Center for Primary Care, Boston, MA USA; 4 The University of Texas at Austin, Dell Medical School, Austin, TX USA; 5 South Texas Veterans Health Care System, San Antonio, TX USA; 6 University of Wisconsin School of Nursing, Madison, WI USA; 7 American Medical Association, Chicago, IL USA

**Keywords:** Hospitalists, COVID-19, Burnout, Workload, Safety

## Abstract

**Background:**

Hospitalist physician stress was exacerbated by the pandemic, yet there have been no large scale studies of contributing factors.

**Objective:**

Assess remediable components of burnout in hospitalists.

**Participants, Study Design and Measures:**

In this Coping with COVID study, we focused on assessment of stress factors among 1022 hospital-based clinicians surveyed between April to December 2020. We assessed variables previously associated with burnout (anxiety/depression due to COVID-19, work overload, fear of exposure or transmission, mission/purpose, childcare stress and feeling valued) on 4 point Likert scales, with results dichotomized with the top two categories meaning “present”; burnout was assessed with the Mini Z single item measure (top 3 choices = burnout). Quantitative analyses utilized multilevel logistic regression; qualitative analysis used inductive and deductive methods. These data informed a conceptual model.

**Key Results:**

Of 58,408 HCWs (median response rate 32%), 1022 were hospital-based clinicians (906 (89%) physicians; 449 (44%) female; 469 (46%) White); 46% of these hospital-based clinicians reported burnout. Work overload was associated with almost 5 times the odds of burnout (OR 4.9, 95% CIs 3.67, 6.85, p < 0.001), and those with anxiety or depression had 4 times the odds of burnout (OR 4.2, CIs 3.21, 7.12, p < 0.001), while those feeling valued had half the burnout odds (OR 0.43, CIs 0.31, 0.61, p < 0.001). Regression models estimated 42% of burnout variance was explained by these variables. In open-ended comments, leadership support was helpful, with “great leadership” represented by transparency, regular updates, and opportunities to ask questions.

**Conclusions:**

In this national study of hospital medicine, 2 variables were significantly related to burnout (workload and mental health) while two variables (feeling valued and leadership) were likely mitigators. These variables merit further investigation as means of reducing burnout in hospital medicine.

**Supplementary Information:**

The online version contains supplementary material available at 10.1007/s11606-023-08309-x.

## Introduction

COVID-19 has been a stressor for healthcare workers like no other. Hospitalists in particular carried a large share of the load during the pandemic by caring for many acutely ill patients with uncertain prognoses in the context of constantly changing COVID-related guidelines. ^1,2^ Early in the pandemic, stress among hospitalists was in the top 5 of all specialties, approaching that seen in Emergency Medicine.^3^ The pandemic significantly impacted physician worklife and wellbeing. Physician burnout increased during COVID-19, ^4,5^ with burnout (which includes emotional exhaustion, depersonalization and lack of sense of accomplishment) in excess of 60% of all practicing physicians by late 2021, the highest levels found since physician burnout has been tracked. In a 2015 survey, 183 academic hospitalists had a lower burnout rate (33%) than 396 general internists in ambulatory care (40%); hospitalists differed from ambulatory clinicians in describing their worklives (with more frequent endorsement of good teamwork, as well as less documentation time pressure and less electronic medical record time spent at home), suggesting there may be unique contributors to worklife in hospital medicine. ^6^

Burnout is associated with intention to leave, intention to reduce hours and suicidal ideation. ^7,8,9^ Stress and burnout have disproportionately impacted certain groups (women, younger faculty).^10^ The American Medical Association’s (AMA) Coping with COVID-19 survey has highlighted issues for critical care physicians and nurses ^8^ and a diverse sample of healthcare workers (HCWs) in numerous work roles; ^10^ the survey has also connected burnout to work intentions among clinicians and nurses. ^7^ However there have been few recent studies of hospitalists, with no large scale studies during the pandemic. (A recent excellent article studied 52 hospitalists at one medical center. ^2^) Most prior studies have been of academic hospitalists, ^11,12^ with little known outside of academic centers. The national survey through the Association of Chiefs and Leaders of General Internal Medicine (ACLGIM) showed differences between hospital medicine clinicians and ambulatory internists, suggesting worklife may be different in hospital medicine. ^6^ A recent paper in JGIM ^13^ described promising worklife interventions in hospital medicine, including improvements in communication (transparent and frequent), wellness resource offerings (related to nutrition and sleep), clear return to work guidelines, increased mental health resources, inpatient use of telemedicine, childcare/elder care resources, and changes in inpatient scheduling, but the evidence base for their use is evolving. Thus, we felt a study of remediable risk factors for burnout in a larger sample of hospital-based clinicians could contribute to the literature.

A recent conceptual framework for hospitalist workforce adaptations ^14^ considered roles of hospitalists during the pandemic, incorporating adaptations employed during the early pandemic, clinical and operational aspects of work, system constraints, and associations with patient and workforce outcomes. We used this framework to refine our understanding of how hospitalist clinicians (defined as physicians and Advanced Practice Clinicians (APCs) in hospital medicine) coped with COVID-19. We hypothesized that use of the framework could highlight actionable changes to prevent, mediate, and reverse burnout among hospitalists.

## Methods

**Figure 1:**
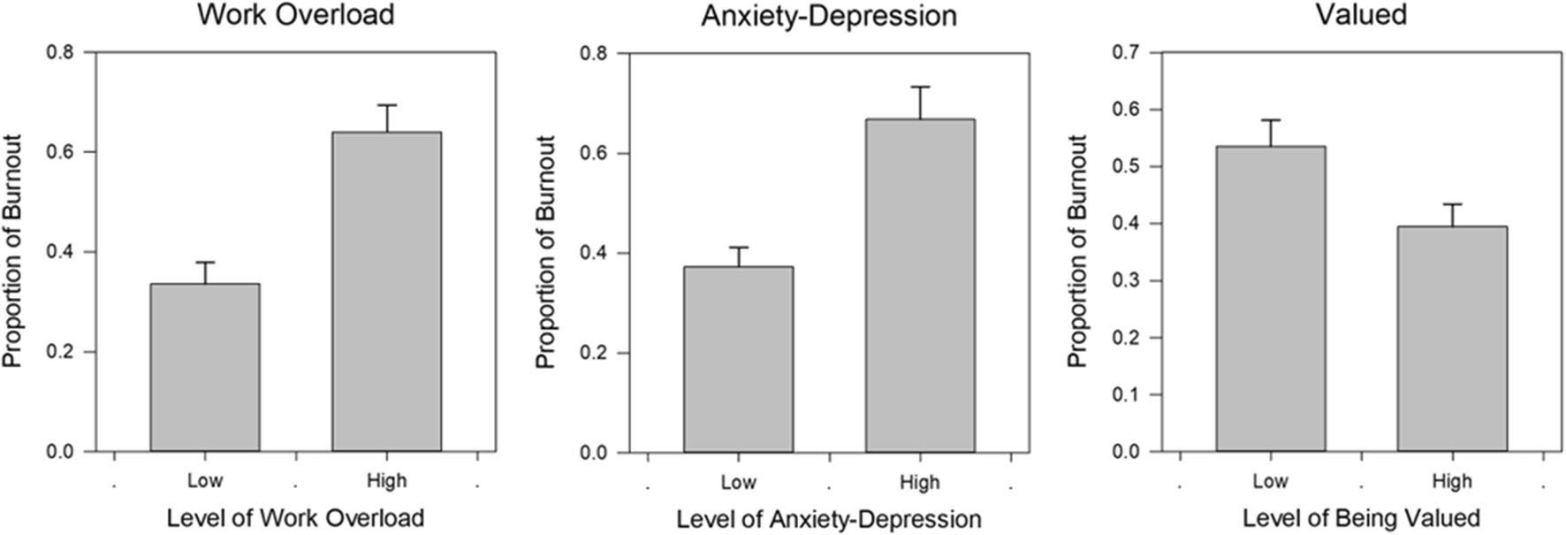
Bar graph of factors related to burnout in hospitalist clinicians. Differences in proportion with burnout in those with and without key worklife variables (work overload, anxiety/depression due to COVID-19 care, and feeling valued), after adjustment.

The previously described Coping with COVID-19 survey ^3,7,10^ involved more than 100 healthcare organizations, many of whom were invited by the AMA to participate. Individual health systems distributed surveys electronically and results were returned to a data lab, Forward Health Group (Madison, WI). Conducted between April of 2020 and March of 2021, the study assessed aspects of worklife (referring to what happens during time one spends at ones job compared with time spent at home) as well as outcomes including satisfaction, burnout, and intent to reduce hours or leave the job. The current study included responses from April 30 until December 12, 2020. Those surveyed (see Coping with Covid survey questions in Appendix, Fig. 1A) included HCWs in varied roles. The current study focuses on those in hospital medicine (physicians and APCs), referred to as hospital-based clinicians. Burnout was measured with the single item Mini-Z assessment (correlated with emotional exhaustion on the Maslach Burnout Inventory ^15^), then dichotomized in the standard manner ^3^ into those who were “burned out” and those “not burned out” (choices c, d and e, each of which mention burnout, represent “burnout”). The author team assessed worklife elements which might be associated with burnout on 4 point scales (see survey in Appendix), usually with the top two choices representing an item or topic being present, including work overload (top two categories = work overload), anxiety/depressive symptoms due to COVID-19 (top two choices = present), fear of exposure to COVID or transmission to loved ones (top two responses = “fear”), mission/purpose (top two categories = present), childcare stress (top two responses = childcare stress present) and feeling valued, a burnout mitigator (top two responses = feeling valued). Cutoffs for dichotomizing were consistent with prior Coping with Covid studies ^10^ and variables selected in part from prior studies (e.g. prior hospitalist surveys ^6^). These single item assessments have performed well in correlations with clinician outcomes. ^3^ Respondents could provide open-ended comments on stressors. To guide analyses, the authors used the conceptual framework for hospitalist workforce adaptations which considered hospitalist activities plus system constraints and innovations. ^14^

### Quantitative Analysis

Univariate statistics and bivariate comparisons were performed to assess associations with burnout, followed by multilevel logistic regressions, controlling for clustering of clinicians within organizations and adjusting for covariates of race and ethnicity, sex and years in practice.

### Qualitative Analysis

Surveys included a free-text prompt, “What else would you like to tell us about your experience during the COVID-19 crisis?” As in other national survey reports, we used a method of examining comments at the survey’s end to learn about respondent’s thoughts and feelings, ^16,17^ adding richness and texture to quantitative findings. To do so, we took the variable most strongly correlated with burnout (work overload) and selected all available comments from those with the highest work overload, and those not at all concerned about work overload, performing a formal content analysis on responses from these two groups to learn the important issues for hospitalist clinicians in favorable vs unfavorable work environments represented by degree of work overload. Comments ranged from four words to three sentences. Using a grounded theory approach, three of the authors (AB, ES, LL) used standard techniques to identify emerging and recurring themes ^18,19^. These authors then met to discuss the major themes prior to applying them to the data. Reviewers thematically indexed and coded the data following principles of content analysis. ^19,20^ After coding, the authors met to review results and reach consensus. Coping with Covid was deemed a quality improvement/program evaluation project exempt from research requirements by the Hennepin Healthcare Institutional Review Board as a low risk, non-interventional study.

## Results

Respondent demographics are summarized in Table 1. There were 58,408 HCWs who responded to this sample from the Coping with Covid survey (with a median response rate of 32%); among these, there were 13,780 physicians and 3705 APCs with data on burnout, with 1022 hospitalist clinicians (906 (89%) physicians; 449 (44%) female, 469 (46%) White, 222 (22%) Asian, 146 (14%) preferring not to identify race or ethnicity; and 514 (50%) in practice <  = 10 years). Forty-six percent of hospital-based clinicians met criteria for burnout (46% of hospitalist physicians and 48% of hospital-based APCs), vs 58% of critical care clinicians, 52% of family medicine clinicians and 49% of infectious disease specialists. Factors associated with burnout in prior Coping with COVID-19 analyses, ^3,7,10^ including fear of exposure, work overload, sense of mission/purpose, and anxiety and depression due to COVID-19-related care, were tested for association with burnout (Table 2). Variables were entered into a regression model (main results in Table 3, Supplemental Table 1A with full model including adjusting covariates). These analyses identified work overload as the strongest correlate of burnout (Odds Ratio 4.9 (95% CIs 3.67, 6.85, p < 0.001). Those with high anxiety or depression from COVID-19-related work had 4 times the odds of burnout (OR 4.2, CIs 3.21, 7.12, p < 0.001), while hospital-based clinicians who felt valued by their organizations had about half the odds of experiencing burnout (OR 0.43, CIs 0.31, 0.61, p < 0.001). Figure 1 shows significant differences in these variables by those with and without burnout. Sense of mission and purpose was not a significant factor in the model. The McKelvey Zavoina pseudo R-squared demonstrated that the model explained 42% of variance in burnout.

**Table 1:** Demographics of 1022 Hospitalist Clinicians in Coping with COVID-19 Study

	Frequency (Percent) *N* = 1022
Ethnicity	
Missing	114 (11.15)
Prefer not to answer	146 (14.29)
White/Caucasian	469 (45.89)
Hispanic/Latino	20 (1.96)
Black/African American	19 (1.86)
Asian/Pacific Islander	222 (21.72)
Other (please specify)	32 (3.13)
Sex	
Prefer not to answer	81 (7.93)
Male	487 (47.65)
1Female	449 (43.93)
Non-binary/third gender	5 (0.49)
Years in Practice	
Missing	20 (1.96)
1–5 years	267 (26.13)
6–10 years	247 (24.17)
11–15 years	188 (18.4)
16 -20 years	141 (13.8)
More than 20 years	159 (15.56)
Role	
Physician	906 (88.65)
Advanced Practice Clinician	116 (11.35)
Setting	
Missing	119 (11.64)
Hospital-based: ER or ICU	209 (20.45)
Hospital-based: non-ER, non-ICU	693 (67.81)
Ambulatory-based: COVID care	1 (0.1)

**Table 2:**
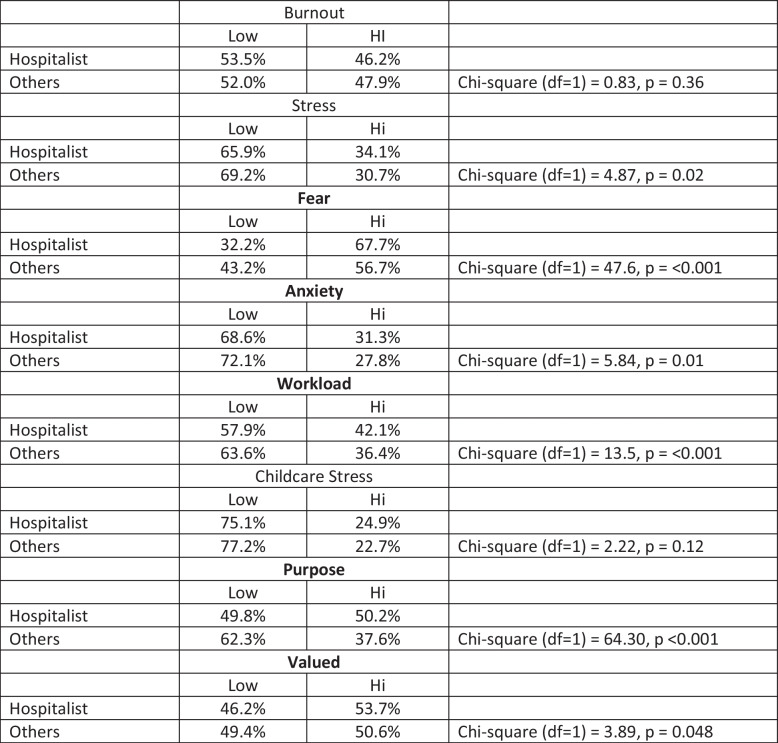
Hospital-based Clinician Worklife Factors Related to Burnout

Chi square testing assesses the significance of differences of a factor’s presence in hospitalist vs other clinicians (e.g. burnout, with no significant difference, or fear of exposure/transmission, which is present significantly more often in hospitalists than others). “Others” refers to the other 16,463 clinicians (physicians and Advanced Practice Clinicians, generalists and subspecialists) in the Coping with Covid study. Variables significantly associated with burnout are highlighted in bold

**Table 3:** Two Level Hierarchical Regression Analyses of Potential Burnout Predictors Among 1022 Hospitalist Clinicians in the Coping with COVID-19 Dataset ^a^

		Odds Ratio	Std. err	Z	*p*	95% CI	
Fear	High	1.124	.2141	0.61	0.539	.7739	1.633
**Workload**	High	4.914	.8925	8.77	0.000	3.44	7.015
Childcare stress	High	1.448	.3001	1.79	0.074	.9649	2.174
**Anxiety/Depression**	High	4.790	.9742	7.70	0.000	3.215	7.136
*Valued*	High	.470	.0862	-4.12	0.000	.328	.6734
Mission/Purpose	High	.8010	.1464	-1.21	0.225	.5597	1.146

McKelvey & Zavoina-Pseudo-R2 (fixed & random effects) = 0.42

Intra-Class Correlation (Level 2) = 0.049

^a^Adjusting covariates were ethnicity, sex and years in practice. Factors that are associated with increased odds of burnout (workload and anxiety/depression) are indicated in bold, while the one associated with a reduced odds of burnout (feeling valued) is noted in italics

*Qualitative comment analysis* (see Consort Diagram, Supplementary Fig. 2A).

**Figure 2:**
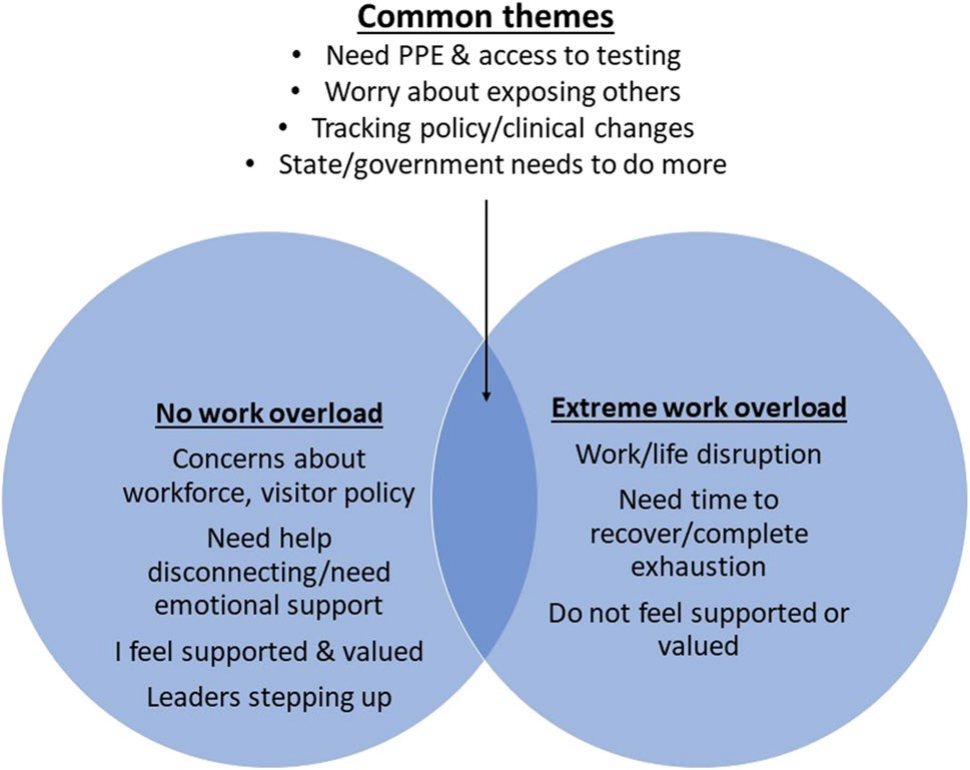
Venn diagram of worklife themes in qualitative analysis of hospital based clinicians with high work overload vs no work overload.

Approximately 55% of hospital-based clinician respondents included comments (517 total comments, after removing n/a or no response cells). The team extracted and reviewed open-ended comments from respondents on the extreme ends of the work overload scale, that is those not experiencing any work overload (*n* = 107 with comments representing those respondents with no work overload) or those experiencing work overload to a great extent (*n* = 109 representing those respondents with extreme work overload). There were 10 total themes identified, with 3 unique to the extreme work overload group, 4 unique to those with no overload, and 4 shared between both groups (see Fig. 2). The “extreme work overload” group had 3 unique themes emerge: 1) work/family balance disruption, 2) time needed to recover from work-related exhaustion, and 3) not feeling valued or supported by leadership or administration. Themes identified as unique to the “no work overload” group included 1) concerns about hospital visitor policies, 2) needing emotional support, 3) feeling valued, and 4) support by leadership. When feeling valued was present, it included comments such as, “I work at an institution that has been prepared for the pandemic, is proactive in equipping us with what we need, and is good at appreciating us”, and “I appreciate the planning that went into preparing for COVID-19 and the flexibility in responding to changing workload and environment.” Meanwhile, comments about leadership in the extreme work overload group were largely negative: (“(I) do not feel supported by our administration…they have taken away a number of resources to help in patient care and expect us to do more with less”), while in the no work overload group, clinicians had positive comments about their leadership (“I feel supported and safe at work. The hospital and team leadership work hard to ensure we are safe.”). Table 4 includes representative quotes from each group related to feeling valued and leadership support. Both groups (no work overload and extreme work overload), shared similarities in their comments, with 4 common themes: 1) concerns about safety (access to personal protective equipment (PPE) and COVID-19 testing); 2) worry about exposing others (e.g. family at home); 3) challenges of keeping track of constantly changing policies; and 4) sentiments that the state or government needs to do more to address the pandemic. Figure 2 shows overlapping and non-overlapping themes from these groups.

**Table 4:** Themes in Hospitalist Clinicians (Physicians and Advanced Practice Clinicians) with No Work Overload and Extreme Work Overload in the Coping with COVID-19 Study

	Work Overload	
Theme	NO (Not At All)	EXTREME (To a Great Extent)
Feeling valued	I work at an institution that has been prepared for the pandemic, is proactive in equipping us with what we need, and is good at appreciating us It’s great to be a part of the team helping out. Not working is harder than working I appreciate the planning that went into preparing for COVID and the flexibility in responding to changing work load and environment	I don’t feel valued by the hospital admin. It was made progressively more difficult for us to obtain our daily mask rations as time went on (pick up site moved progressively further from inpatient units and entry points), one of our work rooms was taken away despite increased work load… It seems the system values money more than lives of providers and this becomes increasingly obvious with every cut The approach to mandating increased hours, increased days for extended periods of time with zero regard to our personal /family life and obligation was a slap in the face. Our underlying value was made VERY clear…
Leadership support	I feel supported and safe at work. The hospital and team leadership work hard to ensure we are safe There was a great collaboration between different departments. We were fast to identify the gaps and start addressing them… The leadership team were extremely supportive Good support from colleagues and institution Our program has been remarkably transparent and informational throughout this time which has been essential to my colleagues feeling supported I have learned what great leadership there is in this organization. The transparency, regular updates, & opportunity to ask questions has made this situation easier to deal with. Sometimes, it is less stressful to be a(t) work than in the community	Do not feel supported by our administration. They have taken away a number of resources to help in patient care and expect us to do more with less I have felt completely unsupported and expendable to my organization Administration keeps sending ridiculous emails inquiring about our health and offering various services. However they have done absolutely nothing to decrease our workload and help our burden at work. In fact they have done the exact opposite. They understaffed us and made us work longer and harder hours, in a time when donning isolation gear, and more obstacles to care exist

## Discussion

In this national study of 1022 hospital-based physicians and APCs during the early stages (April 2020-December 2020) of the pandemic, we applied a conceptual framework for explaining and mitigating burnout in this important population of healthcare workers. Hospital medicine had unique challenges during the pandemic, as did other groups managing acutely ill patients, including emergency medicine, critical care and infectious disease clinicians. In our sample of hospital-based clinicians, close to half were burned out. The results were both distressing and uplifting. Hospitalists shared significant challenges and lack of support as well as innovative opportunities for improvement and even numerous success stories seen in Table 3 (great teamwork, transparent communication with regular updates, feeling safe, and supportive leadership). The most highly correlated variables with burnout were work overload, and anxiety and depression due to COVID-19-related care, which, if confirmed in prospective trials, would offer opportunities to address burnout through 1) defining what a reasonable workload is at the individual level, then developing means for real-time adaptation of workload to individual and team capacity, and 2) building in resources for on-site (accessible) and off-site (more confidential) mental health resource availability. Finally, the importance of feeling valued was demonstrated in this group of clinicians, as it has been shown to be meaningful in other HCWs  and clinicians. ^3^ How to promote feeling valued in hospitalist clinicians is a point for investigation. This large scale, data driven, model-validating study (to our knowledge, the largest study of hospital medicine in the past decade) during the “stress test” of the pandemic provides direction for needed study and action, as there are few such surveys to rely upon to guide current worklife interventions in hospital medicine, and burnout rates in medicine remain high as the effects of the pandemic unfortunately continue to linger.

Work overload is significantly associated with higher burnout. The comments and context of stressed clinicians bring urgency to the need for medicine to join other disciplines that measure workload and reduce work when workers are tired or stressed (e.g., as in the airline industry). As staffing shortages grow, ^21,22^ there is a pressing need to evaluate work structure and culture, right-size workload for capacity of those performing it, and ensure adequate numbers of clinicians to account for those who cannot work because they or their families are sick. In this late stage of the pandemic, thoughtful approaches to workload modulation can be implemented and developed, and then held in reserve for the future should more challenging times return.

Meanwhile, feeling valued was a striking protective factor which was significantly associated with lower burnout even in the context of system constraints such as PPE shortages, changing treatment guidelines and isolation requirements. This offers the opportunity for “quick wins,” allowing organizational leadership to assess what matters most to their workforce in terms of feeling valued, and then rapidly responding. As noted, making people feel valued is described as not just appreciating people; it also encompasses being responsive to workload, and giving people the resources they need to do their jobs. While sense of mission and purpose was present more often in hospital-based vs other clinicians (Table 2), it was not significant in its relationship to burnout in regression models. This variable could be pursued in future studies as a factor of potential importance.

The open-ended comments focused attention on leadership: leadership has impact, and organizational leadership makes a difference, as in work by Dyrbye and colleagues. ^23^ Leadership could be a moderator of burnout from work overload and anxiety by changing the impact of stressors if leaders are engaged and responsive and given leeway by their organizations to do so. Clearly, there are challenges to how a system can address a time when work overload is endemic; involving workers in decision making about this, and customizing workloads to individuals’ abilities to take work duties on, may provide a sustainable way forward. Transparent communication was frequently noted as a specific way in which leadership was supportive. The Hospitalist Well-Being Advocates Toolkit ^24^ from the Society of Hospital Medicine portrays recommendations for leaders for excellent leadership and for leader self-care. Our data suggest listening carefully to HCWs and implementing support may relate to improved satisfaction and thus to retention when future crises occur.

Anxiety and depression related to COVID-19 was the other variable significantly associated with burnout. While the data promote system changes to reduce mental health stressors, there is also a role for on- or off-site care and ready access to mental health resources for alleviation of symptoms. COVID-19 care is stressful, even with good protection, and it is ideal that mental health providers are immediately and confidentially available. Rush University’s wellness consult service, with mental health leaders rounding on frontline staff, is a powerful example of being there for clinicians with “in the moment” care. ^25^ Other examples include coaching to reduce burnout ^26 ^as well as peer support ^27^ and buddy programs. ^28^ Transparent communication from leaders may also mitigate clinician anxiety.

We expanded the conceptual model of hospitalist function and outcomes to account for these variables (workload, mental health symptoms, feeling valued, and leadership, see Appendix Fig. 3A for final model). This model can be tested for its validity in guiding healthcare interventions to improve worklives of hospital-based clinicians.

Our study has several limitations. The study used a convenience sample of healthcare institutions during the rapidly evolving early phase of the pandemic, lacking details around institutional data (e.g. academic vs non-academic, or geographic location). Childcare stress was assessed and while it did not have a statistically significant relationship here (OR 1.5, *p* = NS), this should be reassessed in future work, as prior studies have demonstrated its importance. ^29^ Data were collected over less than one year, and the lingering effects of the pandemic were not assessed. Our convenience sample may not be representative of all clinicians, and we have little knowledge of non-respondents. However, our response rate is considerably higher than many national physician surveys. ^4^ The differential impact of COVID-19 on HCWs in varying roles was not tested. Some organizations were impacted more than others due to different infection rates, resources and local factors which could have affected the findings. Work overload is a subjective assessment, though it has been validated in part through the question from which it was adapted on (lack of) work control, a known predictor of stress and burnout. Finally, several ordered categorical survey items were dichotomized to improve clarity (e.g., percent burned out) which may have decreased sensitivity in seeking variables associated with these outcomes.

Future studies should address variables identified as being critically important by our survey’s hospital medicine respondents: work overload, work-related anxiety/depression (in this case, due to COVID-19), feeling valued, and attention to these issues by leadership. Whether remediating these factors, as suggested by other current study teams ^30,31,32,33^ will lead to improved and sustainable work conditions with lower burnout remains to be tested in prospective trials.

Further identifying and confirming the factors that lead to distress and burnout, and the impacts of remediating these factors, will be important next steps in addressing the worklives and wellness of our nation’s hospital-based clinicians.

### Supplementary Information

Below is the link to the electronic supplementary material.

Supplementary file1 (DOCX 546 KB)

## Data Availability

Data may be available from the authors on request.
