# Supplementary material for: Burnout Among Hospitalists During the Early COVID-19 Pandemic: a National Mixed Methods Survey Study
Source: J Gen Intern Med. 2023 Jul 28;38(16):3581–8. doi: 10.1007/s11606-023-08309-x (PMC10713906; doi:10.1007/s11606-023-08309-x)
Supplement: Supplementary file 1 — Supplementary file1 (DOCX 546 KB) [file 11606_2023_8309_MOESM1_ESM.docx]

Supplement:

Supplemental Table 1A. Full Burnout Model with Adjusting Covariates (see below)

Footnote for supplemental table 1A above. Significant factors in the burnout model are highlighted in bold. Note sample of 893 hospitalist clinicians is less than the 1022 in full sample due to missing responses in some of the variables.

Supplemental Figures:

1A. Survey items

2A. Consort diagram

3A. Conceptual model

Supplemental Figure 1A: AMA Coping with Covid Survey Questions

**Coping with COVID-19 for Caregivers Survey:**

A brief survey designed to assess the impact of COVID-19 on clinical and nonclinical staff. This survey should take less than 3 minutes to complete and can be deployed every 4-6 weeks to provide ongoing monitoring.

1. **The stress I experienced today is**
   1. Minimal
   2. Modest
   3. High
   4. Very high
2. **I worry about exposing myself and my family to COVID**
   1. Not at all
   2. Somewhat
   3. Moderately
   4. To a great extent
3. **Due to the impact of COVID 19, I am experiencing the following:**
   1. Anxiety or depression [not at all, somewhat, moderately, to a great extent]
   2. Work overload [not at all, somewhat, moderately, to a great extent]
   3. Concerns about childcare [not at all, somewhat, moderately, to a great extent]
4. **How would the following improve your ability to sustain through the COVID crisis?**
   1. Staff or colleague support for inbox, documentation, and order entry [not at all, somewhat, moderately, to a great extent, N/A]
   2. Healthy food available at all hours [not at all, somewhat, moderately, to a great extent, N/A]
   3. Personal access to mental health care [not at all, somewhat, moderately, to a great extent, N/A]
5. **Being part of the COVID-19 response has increased my sense of meaning and purpose**
   1. Not at all
   2. Somewhat
   3. Moderately
   4. To a great extent
6. **I feel valued by my organization**
   1. Not at all
   2. Somewhat
   3. Moderately
   4. To a great extent
7. **Using your own definition of “burnout,” please choose one of the answers below:**
   1. I enjoy my work. I have no symptoms of burnout
   2. I am under stress, and don’t always have as much energy as I did, but I don’t feel burned out.
   3. I am beginning to burn out and have one or more symptoms of burnout, e.g. emotional exhaustion
   4. The symptoms of burnout that I’m experiencing won’t go away. I think about work frustrations a lot.
   5. I feel completely burned out. I am at a point where I may need to seek help
8. **What else would you like to tell us about how your experience during the COVID 19 crisis?** (Note: Your anonymous answers may be viewed by your institution or practice manager) If you are feeling severe distress, please contact your local Employee Assistance Plan, mental health resource or the national suicide prevention helpline at 1-800-273-8255.
   1. [[free text]]

Supplemental Figure 2A – Consort diagram


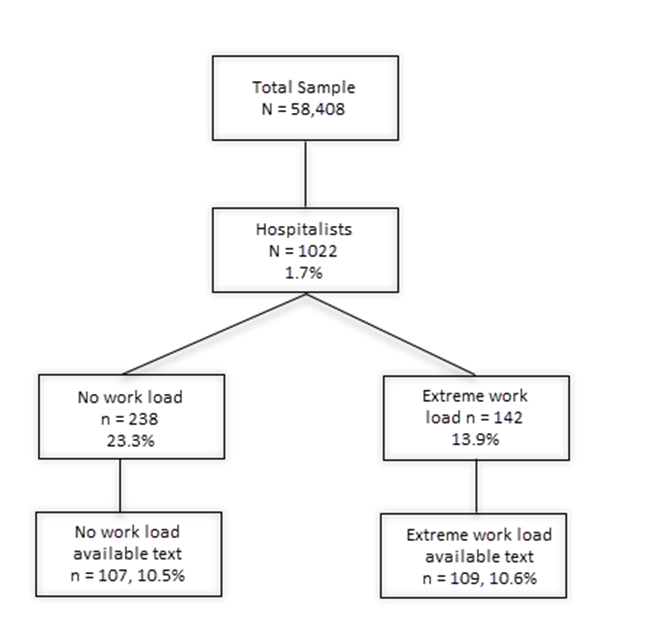


Figure legend: Consort diagram of 58,408 healthcare workers in Coping with Covid study between April and December 2020. Surveys were sent to 303,142 individuals; 17,485 of those who replied to the burnout question were clinicians (physicians and APCs), and 1022 of these clinician respondents were hospitalist clinicians (physicians and APCs). “Available text” refers to those hospital-based clinicians in high workload vs no work overload groups with available text comments for analysis.

Supplemental Figure 3A. Conceptual model of worklife adaptations for burnout reduction in hospital-based clinicians during the COVID-19 pandemic.


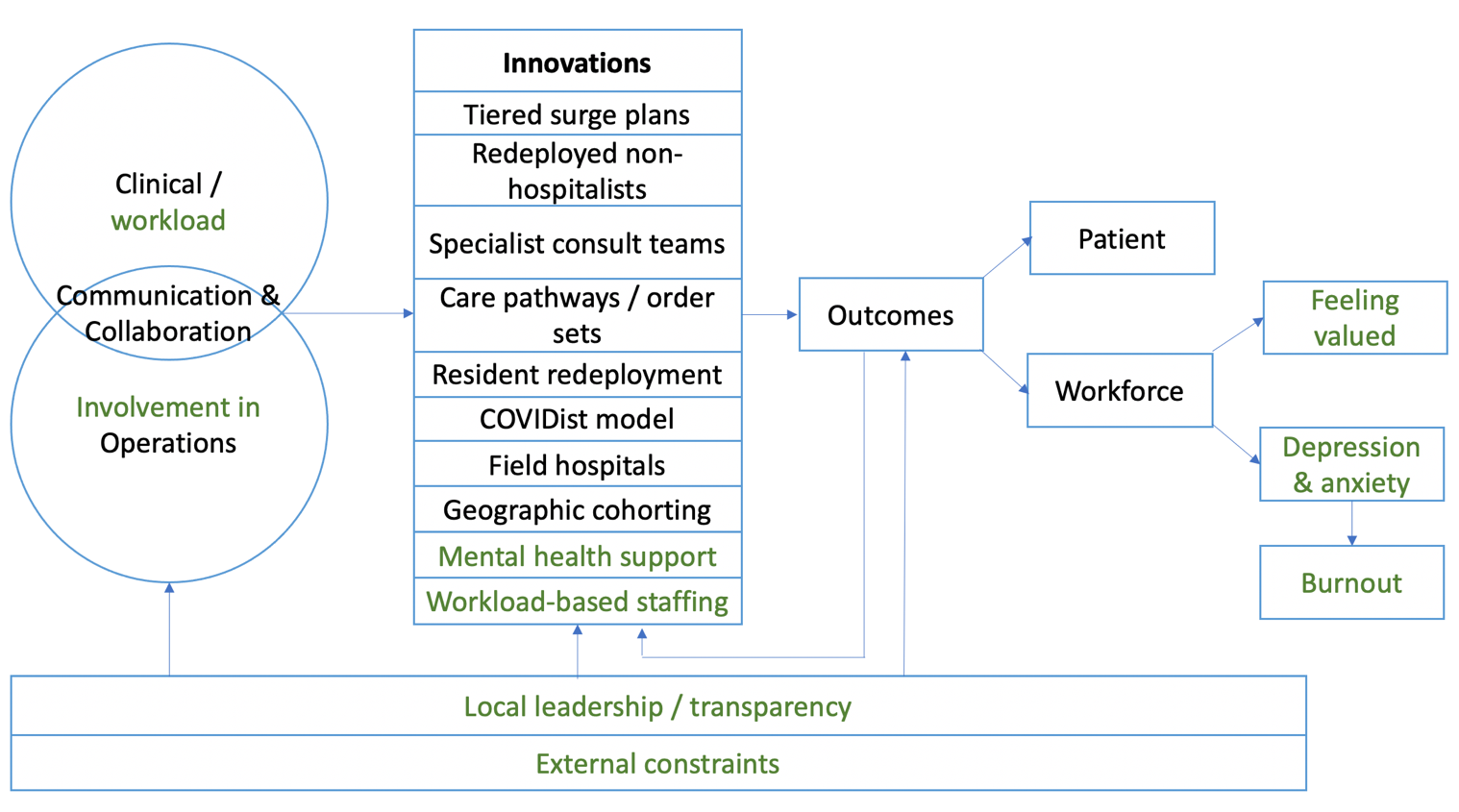


Figure Legend:

This figure is an update to a previously published conceptual model of hospital medicine workforce planning, deployment, and adaptation.^14^ The original model was developed based on data collected through the Hospital Medicine Reengineering Network (HOMERuN) Collaborative Workforce Planning workgroup. These data include focus group and survey data collected from April 2020 through the spring of 2021. Original model components are demonstrated in black font. Refinements to the model made during this analysis based on survey responses are listed in green.

**Clinical / workload**: In the original model, “clinical” referred to the clinical work performed by hospitalists, as well as the way that clinical work is structured – e.g., number and duration of shifts, number and types of hospitalized persons seen during a shift. The term “workload” was added in this updated conceptual model, as workload is a critical aspect of clinical work that was particularly relevant to hospitalist perceptions of burnout.

**Involvement in operations** refers to how work is organized throughout the hospital, and how initiatives are undertaken to change or improve how that work is organized. For example, this might include processes for admitting persons to the hospital, how diagnostic testing is prioritized and performed, interactions between consulting and admitting services, or interprofessional team meeting logistics. The term “involvement in” was added to the conceptual model because of the greater degree of hospitalist involvement in operations since the pandemic, and the mitigating effect that operational involvement can have on burnout.

**Communication and collaboration** refers to the bridging functions performed between those individuals in operations who are organizing the work, and those who are performing the work at the clinical front-lines. Specifically, it focuses on communication between those groups, and how those groups collaborate to develop the best operational strategies to support the front-line clinical work.

**Innovations** refers to new ways of doing things in response to the covid pandemic. All of the examples listed in this column are taken from examples reported in the HOMERuN network, or in written-in survey responses.

**Outcomes** refer to any outputs from the system in which hospitalists work, and are further stratified into patient outcomes and workforce outcomes. Based on the survey results, workforce outcomes were further stratified into feeling valued, a positive outcome, versus depression and anxiety, which in turn lead to burnout. These later outcomes are negatie workforce outcomes.

**Local leadership / transparency** refers to attributes of the leadership in the systems in which hospitalists work. In the original model, this was encompassed in a more general category called “system constraints.” This concept was better specified to refer to transparent sharing of information and decision-making processes and rationales based on survey responses.

**External constraints** refers to factors outside of the healthcare system that influence how hospitalists work, the types of innovations that are possible, and the outcomes of the system. Examples might include availability of personal protective equipment or vaccine availability.
